# Complex beam shaping based on an equivalent q-plate system and analysis of its properties using digital holography polarization imaging

**DOI:** 10.1038/s41598-017-02973-w

**Published:** 2017-06-05

**Authors:** Ching-Han Yang, Andy Ying-Guey Fuh

**Affiliations:** 1 Department of Photonics, National Cheng Kung University, Tainan, 701 Taiwan; 2 Department of Physics, National Cheng Kung University, Tainan, 701 Taiwan; 3 Advanced Optoelectronic Technology Center, National Cheng Kung University, Tainan, 701 Taiwan

## Abstract

In this study, we generate various complex beams carrying angular momentum (AM) by using a programmable beam shaping system to mimic typical q-plates. When a circularly polarized wave is incident onto the system, the emerging beam reverses its spin handedness and obtains a spatial phase factor. This phase factor can be engineered by designing a computer-generated hologram (CGH) and applying it to a spatial light modulator (SLM) to produce a beam with controllable spatially distributed orbital angular momentum (OAM) density. To determine the properties of the generated fields, we combine digital holography (DH) with the beam shaping system to yield visualizations of the beam intensity, phase, and AM distributions over the transverse plane at different propagation distances. Comparisons of the theoretically and experimentally obtained results show good qualitative agreement. This study advances our understanding and interpretation of AM characteristics produced by a programmable q-plate-like system.

## Introduction

It is well known that photons carry not only linear momentum but also angular momentum (AM)^1^. In paraxial approximation, the total angular momentum (TAM) can be decomposed into the spin angular momentum (SAM) and the orbital angular momentum (OAM)^2, 3^. It is believed that the former is related to the polarization of light, while the latter is related to the phase profile of light^1^. For an elliptically polarized light beam, each photon carries SAM, as given by *σħ*, where *σ* = ±1 for the right- and left-handed circularly polarized beams. For a light beam having a helically distributed phase front exp(i$\ell$ *ϕ*), each photon carries OAM given by $\ell$ *ħ*, where $\ell$ is the topological charge (TC). Over recent decades, it has been observed that a trapped particle can either spin or orbit depending on whether SAM or OAM is transferred to the particle^4, 5^. It has also been found that OAM and SAM can be added or subtracted from each other^6^. Moreover, coupling these two momenta occurs when light is tightly focused through a lens with a high numerical aperture^7^ or passes through an inhomogeneous and anisotropic medium^8, 9^. Apart from optical tweezers, OAM-carrying light has been found to have potential use in optical communication^10^ and remote sensing^11^.

A number of methods for generating OAM-carrying light have been proposed, such as using spiral phase elements^12^, computer-generated holograms (CGHs)^13^, and liquid crystal (LC) q-plates^14–16^. Of these methods, q-plates, which are half-wave plate retarders with inhomogeneous patterned principal axes on the transverse plane, are especially attractive due to their ability to transfer OAM to light^9^ and to generate vector beams^15^, and their potential use in high-speed communication^17^. To date, most q-plates are LC devices based on the photo-alignment technique^14, 16^. Also, the q value, which is defined as the changing rate of the principle axes of LCs, remains fixed in the fabrication design and thus these devices are not flexible for use as spatial light modulators (SLMs). More recently, an equivalent system of q-plates was proposed that mimics typical q-plates^18^. This proposed imitated q-plate system has the benefit of leaving out the fabrication of q-plates and thus has more degrees of freedom in the real-time manipulation of light. However, the OAM of generated fields did not be investigated adequately in the work^18^.

In this paper, we extend the method proposed by Moreno *et al*.^18^ for generating various OAM-carrying complex beams. Additionally, we combine digital holography (DH) with the beam shaping system. DH is a well-developed technology that allows for the non-invasive acquisition of both light intensity and phase information^19, 20^. Therefore, it enables the study of the structure and propagation characteristics of generated beams, such as their intensities, phases, and OAM density distributions. We present both measurement and theoretical results in this paper. Compared with earlier work on so-called meta-q-plates^16^, our system has the advantage of being fabrication-free. Moreover, our DH imaging results improve our understanding and interpretation of the characteristics of the OAM produced by a programmable q-plate-like system.

## Results

### Experimental setup

**Figure 1:**
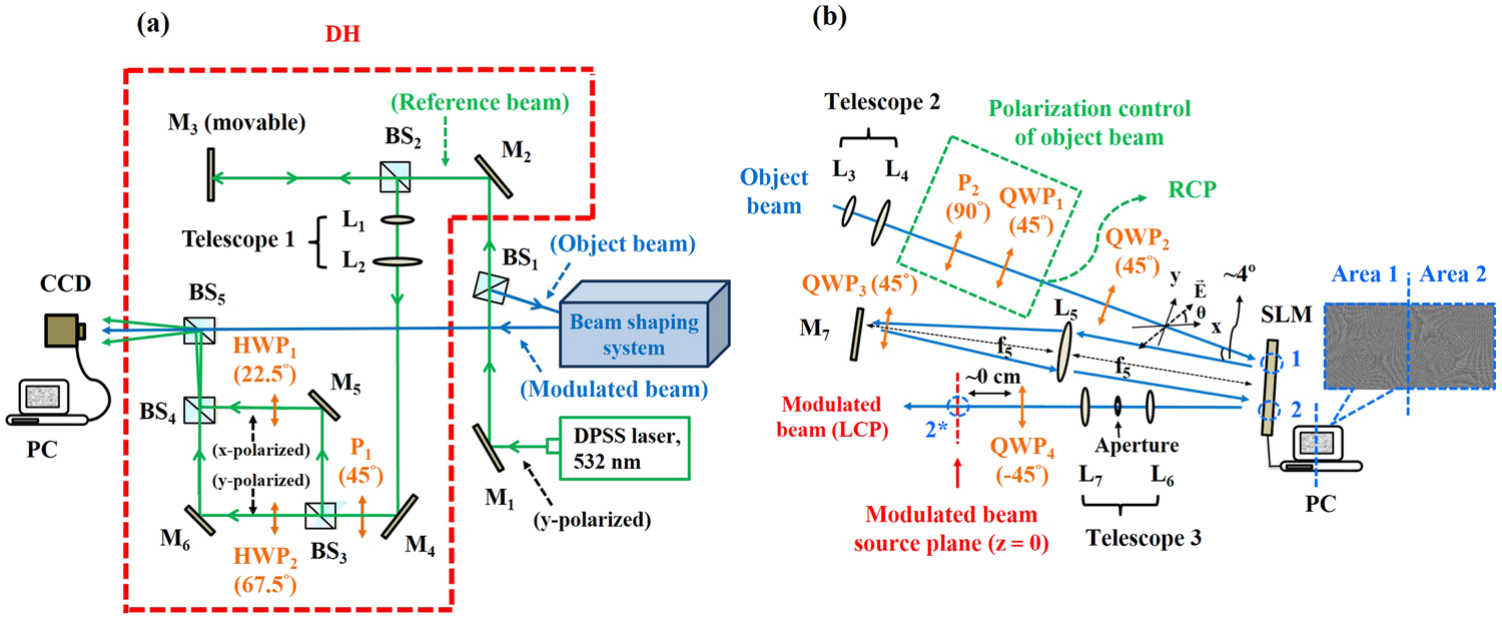
(**a**) Experimental setup of the imitated q-plate beam shaping system combined with DH. (**b**) Detailed schematic of the imitated q-plate system arrangement. L: lens, P: polarizer, HWP: half-wave plate, QWP: quarter-wave plate, M: mirror, BS: non-polarizing beam splitter with 50:50 split ratio. All the orientation angles of the slow (optical) axis of wave plates and the transmission axis of polarizers with respect to the x-axis are also given in the figure. The inset at the right-top corner of (**b**) shows an example of simultaneously displaying two blazed holograms side by side on the SLM. The distance between the modulated beam source plane, denoted as plane 2*, and the CCD plane is about 24 cm.

Figure 1 shows the experimental setup of the imitated q-plate beam shaping system combined with DH. Figure 1(b) gives a detailed schematic of the beam shaping system arrangement, which is similar to our previous setup for generating vector beams^21^. As shown in Fig. 1(a), a diode-pumped solid-state (DPSS) laser beam (Verdi, *λ* = 532 nm) with vertically polarized state is split into reference and object beams upon passing through a non-polarizing beam splitter BS_1_. The object beam is modulated by the beam shaping system, while the reference beam is superimposed onto the modulated beam. To obtain significant interference visibility, the position of mirror M_3_ is adjustable to obtain nearly equal optical paths between the object and reference beams. The reference beam is filtered and collimated via telescope 1, which consists of lens L_1_ (*f*_1_ = 15 mm) and lens L_2_ (*f*_2_ = 150 mm), and then passes through a polarizer P_1_, which is used to generate a linearly polarized beam with its polarization making an angle of 45° with respect to the x-axis. Next, the reference beam is further split into two sub-reference beams when passing through BS_3_. One of the two reference beams becomes x-polarized after it passes through the half-wave plate HWP_1_ with its slow axis making an angle of 22.5° with respect to the x-axis; while the other becomes y-polarized after passing through HWP_2_ with its slow axis making an angle of 67.5° with respect to the x-axis. Finally, two sub-reference beams are incident onto a charge-coupled device (CCD) camera (Newport LBP-4-USB) as they pass through BS_5_. It is noted that optical elements of M_5_, M_6_, BS_3_, and BS_4_ constitute a rectangular structure, and this ensures that the two sub-reference beams have nearly the same optical paths. Otherwise, the interference visibility made by the x-polarized components of the reference and modulated object beams is very different from that made by the y component, and such an effect could cause inaccurate DH imaging results. In addition, both M_5_ and M_6_ are slightly tilted such that the two sub-reference beams are obliquely incident on the CCD camera at a small incidence angle, while the modulated beam is incident normally on the CCD camera. On the other hand, as shown in Fig. 1(b), the object beam is first filtered and collimated via telescope 2, which consists of lens L_3_ (*f*_3_ = 15 mm) and lens L_4_ (*f*_4_ = 150 mm), and then subsequently passes through a polarizer P_2_ with its transmission axis oriented at the y-axis and a quarter wave-plate QWP_1_ with its slow axis oriented at 45° with respect to the x-axis. P_2_ is introduced to generate a y-polarized beam, while QWP_1_ is utilized to generate a right-handed circularly polarized (RCP) beam. Next, the object beam is incident onto the imitated q-plate system, which is a train of optical elements sandwiched between two crossed wave plates QWP_2_ and QWP_4_, wherein the slow axis of QWP_2_ is oriented at 45° while that of QWP_4_ is oriented at −45° with respect to the x-axis. Here, we considered the phase-only modulation effect of the SLM, that is, only the x-polarized beam can be phase-modulated while the y-polarized beam cannot^18^. To overcome this problem, we used the double modulation scheme proposed by Moreno *et al*.^22^. The panel of the SLM (Holoeye Photonics, PLUTO-VIS, 1920 × 1080 pixels) is divided into equal areas, and each modulates the x- and y-polarized components of incident light, respectively^21, 22^. In addition, we employed a reflective 4f (*f*_5_ = 75 cm) imaging system to image area 1 of the SLM onto area 2 and inserted QWP_3_ with its slow axis oriented at 45° relative to the x-axis to spatially inverse the x- and y-polarization components, as explained in the supplementary information. As a result, two orthogonal linear polarization components of incident light can be successively phase modulated when passing through different half panels on the SLM in turn. Next, telescope 3, consisting of L_6_ (*f*_6_ = 20 cm) and L_7_ (*f*_7_ = 30 cm), images area 2 of the SLM onto its conjugated plane (2*), which is also close to the QWP_4_. As demonstrated in the supplementary information, light passing through QWP_4_ is identical to the beam emerging from a typical q-plate, and thus we refer it as a modulated beam. In this process, two blazed gratings with an equal but opposite periodicity of 9.5 pixels oriented at 45° relative to the x-axis are respectively added to two designed CGHs displayed on each half of the SLM. The reason for two blazed gratings with opposite periodicity is to compensate the effect of inverse imaging of the 4f imaging system. As such, the first diffraction orders of the x- and y-polarized beams are angularly separated from other orders, which are then blocked using an aperture placed at the focal plane of telescope 3^23^. In addition, the beam width of the encoded field was set to be 250 pixels by spatially modulating the contrast of the grating structures^24^. To record digital holograms, we placed a CCD camera about 24 cm behind the conjugated plane 2*. Finally, we used a computer to perform a numerical reconstruction process based on the diffraction theory to analyze optical information regarding the beam intensity, phase, OAM, and TAM density distributions on different propagation distances.

### Theoretical modeling of the imitated q-plate beam shaping system

The train of optical elements sandwiched between the two orthogonally oriented wave plates QWP_2_ and QWP_4_ in Fig. 1(b) constitute the imitated q-plate. As demonstrated in the supplementary information, the Jones matrix of the imitated system is exactly the same as that of a typically tuned q-plate^9^, as given in equations (1) and (2), respectively. The word “tuned” means that the q-plate is applied at an appropriate voltage so that its optical phase retardation is *π* ^14^. For simplicity, we use the term q-plates in the remaining text.

1 $${{\boldsymbol{M}}}_{imitatedsystem}=(\begin{array}{cc}\cos \,(p) & \sin \,(p)\\ \sin \,(p) & -\cos \,(p)\end{array})$$

2 $${{\boldsymbol{M}}}_{q-plate}=(\begin{array}{cc}\cos \,\mathrm{(2}q\varphi ) & \sin \,\mathrm{(2}q\varphi )\\ \sin \,\mathrm{(2}q\varphi ) & -\cos \,\mathrm{(2}q\varphi )\end{array})$$

where *p* is the phase distribution of the displayed CGH on area 1 of the SLM, *ϕ* is the azimuthal coordinate of the beam, and 2q specifies the TC of the q-plate. We note that the two CGHs displayed on each half on the SLM are opposite in sign, as explained in the supplementary information. A comparison of equations (1) and (2) reveals that the imitated system allows for the real-time configuration of arbitrary q-plates. Therefore, similar to typical q-plates, various high-order cylindrical vector beams can be rapidly generated when the incident beam is a linearly polarized^17, 18^, and conversion of the AM from SAM to OAM can occur when the incident beam is circularly polarized^9, 14, 15^.

### Theoretical description of polarization imaging in digital holography

The method that we used to record and reconstruct information about modulated beams is similar to that used in previous work^25, 26^, which permits the imaging of the light polarization state. In DH, the resulting intensity between two orthogonal linear polarization reference beams and a modulated beam produces a hologram that is subsequently digitalized by a CCD camera and recorded on a computer. The intensity distribution of the digital hologram can be expressed as follows:

3 $$\begin{array}{rcl}{\rm{I}} & = & {|\overrightarrow{O}+{\overrightarrow{R}}_{x}{e}^{j{\overrightarrow{k}}_{x}\cdot \overrightarrow{r}}+{\overrightarrow{R}}_{y}{e}^{j{\overrightarrow{k}}_{y}\cdot \overrightarrow{r}}|}^{2}\\  & = & ({\overrightarrow{O}}_{x}+{\overrightarrow{O}}_{y}+{\overrightarrow{R}}_{x}{e}^{j{\overrightarrow{k}}_{x}\cdot \overrightarrow{r}}+{\overrightarrow{R}}_{y}{e}^{j{\overrightarrow{k}}_{y}\cdot \overrightarrow{r}})\cdot {({\overrightarrow{O}}_{x}+{\overrightarrow{O}}_{y}+{\overrightarrow{R}}_{x}{e}^{j{\overrightarrow{k}}_{x}\cdot \overrightarrow{r}}+{\overrightarrow{R}}_{y}{e}^{j{\overrightarrow{k}}_{y}\cdot \overrightarrow{r}})}^{\ast }\\  & = & {|{\overrightarrow{O}}_{x}|}^{2}+{|{\overrightarrow{O}}_{y}|}^{2}+{|{\overrightarrow{R}}_{x}|}^{2}+{|{\overrightarrow{R}}_{y}|}^{2}+{\overrightarrow{O}}_{x}\cdot {({\overrightarrow{R}}_{x}{e}^{j{\overrightarrow{k}}_{x}\cdot \overrightarrow{r}})}^{\ast }\\  &  & +\,{\overrightarrow{O}}_{x}^{\ast }\cdot ({\overrightarrow{R}}_{x}{e}^{j{\overrightarrow{k}}_{x}\cdot \overrightarrow{r}})+{\overrightarrow{O}}_{y}\cdot {({\overrightarrow{R}}_{y}{e}^{j{\overrightarrow{k}}_{y}\cdot \overrightarrow{r}})}^{\ast }+{\overrightarrow{O}}_{y}^{\ast }\cdot ({\overrightarrow{R}}_{y}{e}^{j{\overrightarrow{k}}_{y}\cdot \overrightarrow{r}})\end{array}$$

where $\overrightarrow{O}$ = ${\overrightarrow{O}}_{x}$ + ${\overrightarrow{O}}_{y}$ represents the modulated beam, ${\overrightarrow{O}}_{x}$ and ${\overrightarrow{O}}_{y}$ respectively represent the x- and y-polarized modulated beams, and ${\overrightarrow{R}}_{x}$ and ${\overrightarrow{R}}_{y}$ respectively represent the x- and y-polarized reference beams. The first fourth terms of equation (3) are zero-order terms; the fifth (seventh) and sixth (eighth) terms are, respectively, the virtual and real image terms of the horizontal (vertical) component of the interference terms; and we have neglected the interference terms between ${\overrightarrow{R}}_{x}$ and ${\overrightarrow{R}}_{y}$, ${\overrightarrow{O}}_{x}$ and ${\overrightarrow{O}}_{y}$, ${\overrightarrow{O}}_{x}$ and ${\overrightarrow{R}}_{y}$, and ${\overrightarrow{O}}_{y}$ and ${\overrightarrow{R}}_{x}$ due to orthogonality. Because an off-axis geometry is employed in the experiment, and thus it yields the two tilted wave fronts ${e}^{j{\overrightarrow{k}}_{x}\cdot \overrightarrow{r}}$ and ${e}^{j{\overrightarrow{k}}_{y}\cdot \overrightarrow{r}}$ for the x- and y-polarized reference beams, respectively, incident on the CCD camera. This makes it possible to separate twin images from zero-order terms when computing the Fourier transform (FT) of the digital hologram^25, 27, 28^. The inverse FT of the filtered spectra results in two filtered holograms containing only virtual image terms of equation (3), as given by the following:

4 $${{\rm{I}}}_{{\rm{i}}}^{{\rm{F}}}={\overrightarrow{O}}_{i}\cdot {({\overrightarrow{R}}_{i}\cdot {e}^{j{\overrightarrow{k}}_{i}\cdot \overrightarrow{r}})}^{\ast }$$

where superscript “F” represents the filtered hologram, and subscript “i” represents the x or y component of light. The next step is to remove the tilted wave front ${e}^{j{\overrightarrow{k}}_{i}\cdot \overrightarrow{r}}$ contained in equation (4); otherwise the reconstruction field is deflected with an angle and thus is not centred in the reconstruction image. In fact, the tilted phase factor can be viewed as an aberration existing in the setup and can be compensated by using the reference conjugated hologram (RCH) method^29, 30^. This method is accomplished by successively recording two holograms, that is, a reference hologram and a signal hologram, and subtracting their common aberration terms on the hologram plane. In the experiment, we first loaded reference CGHs containing no designed phase structure, except for the blazed grating, onto the SLM to generate a blank object beam. This blazed grating is simply used for spatial filtering at the focal plane of telescope 3, as indicated in Fig. 1(b). Then, we obtained a reference hologram by using a CCD camera to record the interference pattern between the blank object beam and the two reference beams. As before, we performed the same filtering process on the reference hologram to obtain two filtered reference holograms, as given by the following:

5 $${{\rm{I}}}_{{\rm{Ref}},{\rm{i}}}^{{\rm{F}}}={\overrightarrow{O}}_{i,blank}\cdot {({\overrightarrow{R}}_{i}\cdot {e}^{j{\overrightarrow{k}}_{i}\cdot \overrightarrow{r}})}^{\ast }$$

where subscript “Ref” indicates the reference hologram and “blank” indicates the blank object beam. In this way, a compensated hologram can be defined as the conjugated phase of equation (5):

6 $${{\rm{\Gamma }}}_{{\rm{i}}}^{{\rm{C}}}\equiv {e}^{\{-j\cdot arg({{\rm{I}}}_{{\rm{Ref}},{\rm{i}}}^{{\rm{F}}})\}}={e}^{j{\overrightarrow{k}}_{i}\cdot \overrightarrow{r}}$$

where superscript “C” indicates compensated, and the operator “arg” denotes the argument of equation (5). If we consider multiplying equation (6) with equation (4) and subsequently dividing the result by the reference beams’ amplitude $|{\overrightarrow{R}}_{i}|$, then we obtain the following:

7 $${{\rm{I}}}_{{\rm{i}}}^{{\rm{C}}}=\frac{{{\rm{I}}}_{{\rm{i}}}^{{\rm{F}}}\cdot {{\rm{\Gamma }}}_{{\rm{i}}}^{{\rm{C}}}}{|{\overrightarrow{R}}_{i}|}={\overrightarrow{O}}_{i}$$

Equation (7) is therefore a corrected hologram containing only the information of the x or y components of the modulated beam. If necessary, we can insert a numerical smooth mask, i.e., an apodized window function, in front of the corrected digital hologram to reduce fringing in the reconstruction plane^31, 32^. Finally, we use a computer to perform the numerical reconstruction of equation (7) within the framework of the angular spectrum method^19^. Then, we can analyze the propagation characteristics of the modulated beams. A detailed description of how to reconstruct the beam intensity, phase, SAM, and OAM density distributions is further outlined in the supplementary information.

### Experimental results

**Figure 2:**
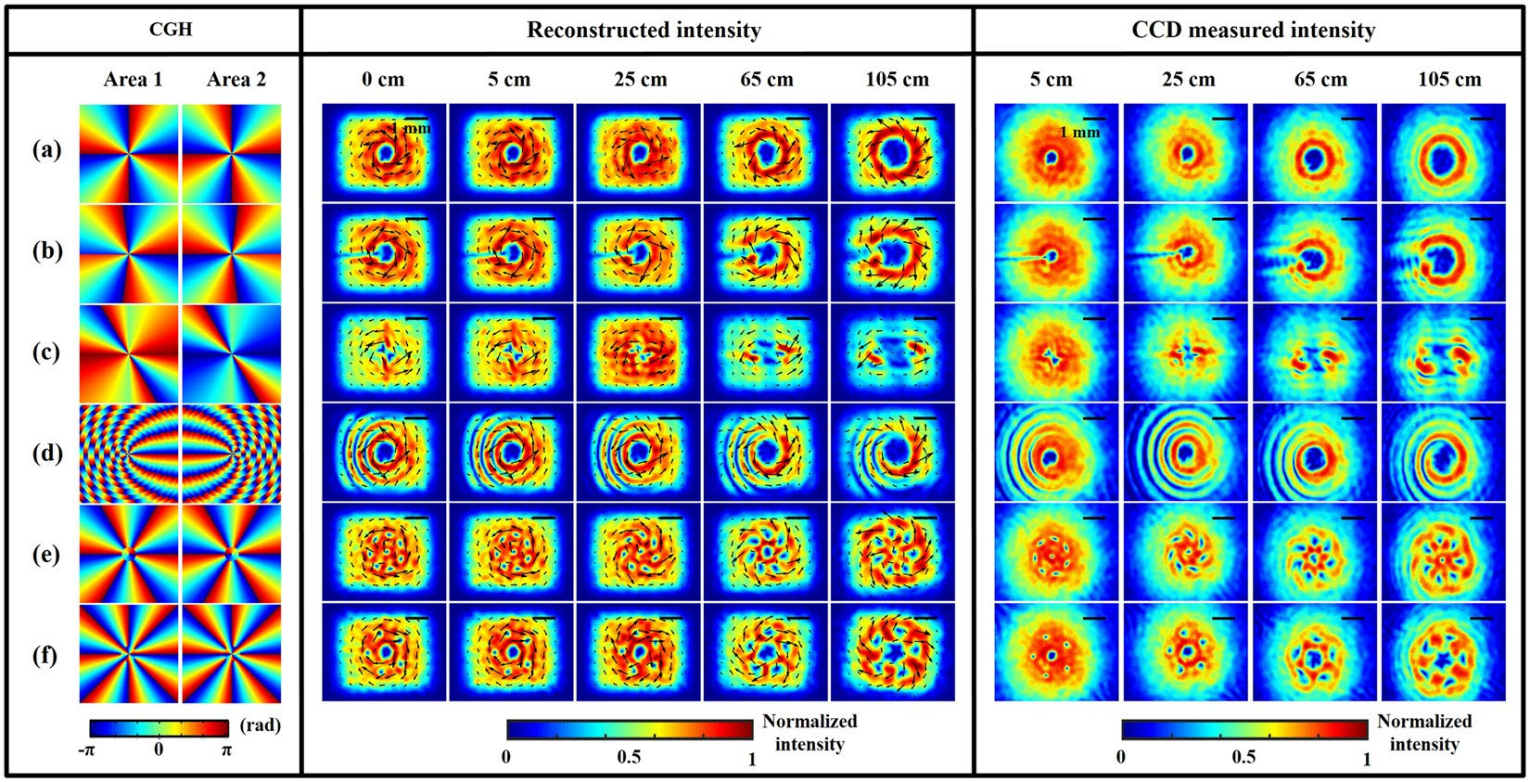
Holograms used to generate complex beams and the corresponding diffraction results along the beam propagation direction. The first block shows the designed CGHs that contain no blazed structure. As explained in the supplementary information, two displayed CGHs in each case are opposite in sign. The second block shows the DH reconstructed results of generated fields from the source plane at z = 0 to other planes at z = 5, 25, 65, 105 cm. The third block shows the intensity patterns captured directly by a CCD camera. (**a**) Case with q = 2, (**b**) case with fraction q = −1.8, (**c**) case with q = −1 in first and third quadrants and q = 3 in second and fourth quadrants, (**d**) case with radially variant q changing from 2 at the center to 8.5 at the border with an interval of 0.5 every 15 pixels, (**e**) case with q = −0.5 for the inner region r ≤ 67 pixels and q = 3 otherwise, (**f**) case obtained by extracting the phase profile from the superposition of LG_0, −3_ and LG_0, −8_ modes. The arrows in the DH results represent the direction of the OFD. The scale bar in the right-top corner in each figure is about 1 mm. The intensity distribution of each figure is normalized to unity.

**Figure 3:**
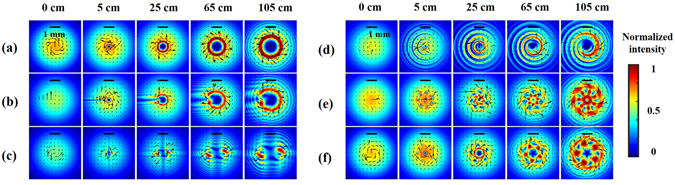
Theoretical diffraction intensities along the beam propagation direction using the angular spectrum method. Each case corresponds to that in Fig. 2. The arrows in all the results represent the direction of the OFD. The intensity distribution of each figure is normalized to unity. The scale bar in the top of each figure is about 1 mm.

**Figure 4:**
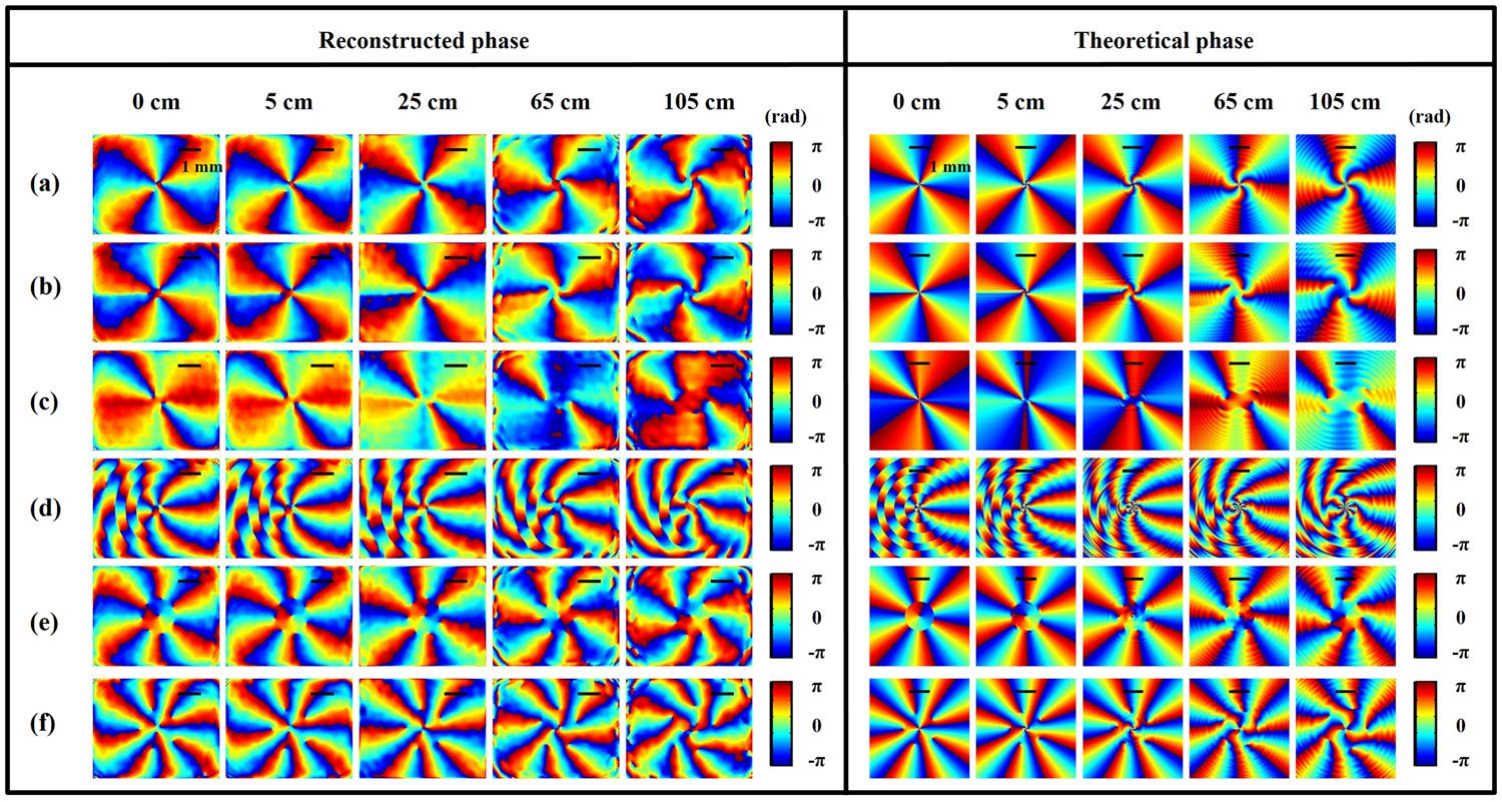
Spatial phase distributions of the x component of light on different cross sections. Left: reconstruction results from DH; right: simulation results. Each case corresponds to that of Fig. 2. The color bar shows the wrapped phase map between −*π* and *π*. The scale bar sketched in all figures is about 1 mm.

The first block in Fig. 2 presents pairs of CGHs that are used in each half of the SLM, in which, for clarity, we have excluded the brazed gratings. A comparison between equations (1) and (2) reveals that the Jones matrix of the imitated q-plate system, in which the CGH with phase *p* displayed on the SLM, is identical to that of a q-plate with TC = 2q. In this way, Fig. 2(a) represents the case of a q-plate with q = 2. Figure 2(b) shows the case with fractional q = −1.8. Figure 2(c) shows the case with q = −1 in the first and third quadrants and q = 3 in the second and fourth quadrants. Figure 2(d) shows the case with a radially variant q changing from 2 at the center to 8.5 at the border, at an interval of 0.5 every 15 pixels. Figure 2(e) shows the case with q = −0.5 at radius r ≤ 67 pixels and q = 3 otherwise. Figure 2(f) shows the phase profile produced by coaxially superimposing two Laguerre-Gaussian (LG) modes, LG_0,−3_ and LG_0,−8_, where the first subscript indicates the radial index and the second indicates the azimuthal index, respectively^33^. The second block in Fig. 2 shows the reconstructed intensities of modulated beams from DH on several planes located at z = 0, 5, 25, 65, and 105 cm from the source plane 2* (see Fig. 1(b)), which we denote as z = 0 cm. Here the arrows in the figures represent the direction of orbital flow density (OFD). For further information on the OFD, please refer to the supplementary information. In order to examine the quality of the images reconstructed from DH, we blocked the reference beam and placed the CCD camera at various distances from the z = 0 plane to directly measure the intensity patterns of the generated beams, as shown in the third block of Fig. 2. A comparison of the results reconstructed by DH and those measured by the CCD camera shows good agreement, except for the difference in shape. The reason for this is that in order to reduce the black-and-white fringes that occur in the reconstruction images, we multiplied the corrected digital hologram, as given by equation (7), by an apodized aperture with transmittance equal to unity in the large central area and which slowly varies from unity to zero at the borders^31^. In contrast, in the situation where an apodized aperture is not used, the imaging fields computed by DH are circular in shape, as given in the Supplementary Fig. S3. To demonstrate the validity of the imitated q-plate system itself, we show the simulation results in Fig. 3 to facilitate comparison with previous results. It can be clearly seen that, except for planes close to z = 0, most of the experimental results coincide well with the simulated results. Inconsistency in the results is due to the fact that when executing the simulation, we assume that a fundamental Gaussian beam is incident onto a virtual q-plate placed at z = 0, where both the polarization components of light are simultaneously modulated. However, in the practical situation, the incident beam is modulated successively by two different halves of the SLM and then passes through QWP_4_, and therefore, we expect that the beam profile will not be exactly the same as the Gaussian beam at the reconstruction plane z = 0. This is the reason why the experimental results disagree with the simulated results at the plane close to z = 0. Moreover, we observe that cases (a)–(d) correspond to results whose intensity profiles are nearly independent of the propagation, whereas cases (e) and (f) are dependent on the propagation. This may be attributed to the local fields that do not satisfy the paraxial wave condition on the transverse plane diffract more strongly away from the beam axis compared to fields that satisfy the paraxial wave condition. Specifically, this happens in a situation where there are evenly distributed phase singularities located at the same radial distance from the beam center (see Fig. 4). As expected, beams that change their intensity profiles as they propagate will finally become stable after propagating for a long distance. To further understand the evolution of intensity profiles, Supplementary Video 1 shows an animation of the evolution of the intensity profiles with propagation.

Figure 4 shows the light spatial phase distributions of the x component, where the reconstruction phase is referred to as the wrapped phase bounded between −*π* and *π*. Here, we omit discussions of the y components on the basis that the y component is simply *π*/2 out of phase with the x component for a LCP wave (recalling that the incident object beam in our experiment is RCP, and thus the output wave is LCP). As we can see, there is very good agreement between the theoretically predicted results and the DH reconstructed results, except that they may differ from an overall constant phase factor, which is unimportant for most purposes. Moreover, all the reconstructed phase distributions resemble those of holograms displayed on the SLM, especially at plane z = 0. This can be understood by considering that when a RCP wave propagates through the imitated q-plate system, whose Jones matrix is given in equation (1), then the outgoing beam becomes LCP and also gains a phase factor, wherein *p* is the phase distribution of the CGH displayed on the SLM. After that, the phase will continue to evolve as the beam propagates in free space. Supplementary Video 2 shows an animation of the phase profiles evolving with propagation.

**Figure 5:**
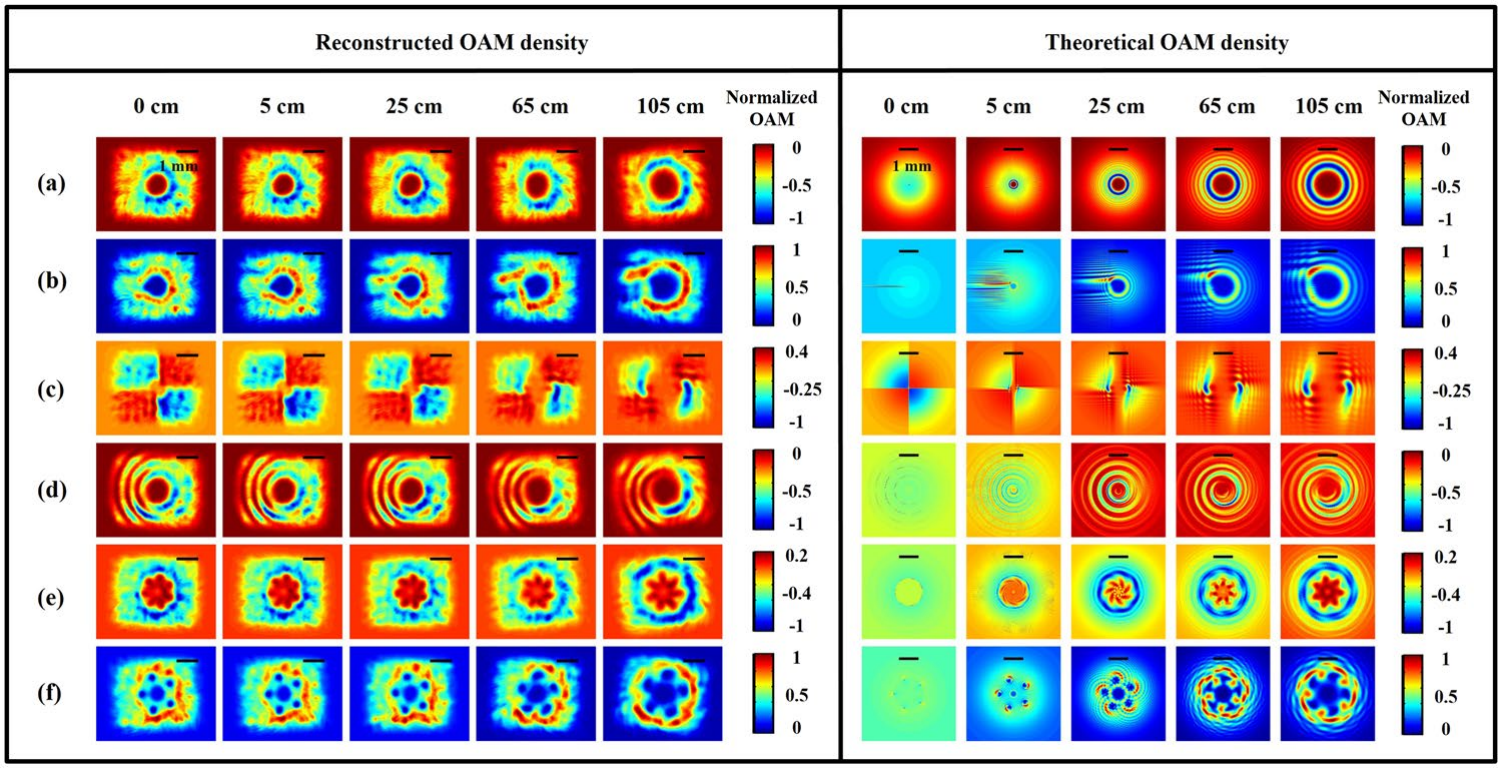
Spatial OAM density distributions of modulated beams on different cross sections. Left: DH reconstructed results; right: simulation results. Each case corresponds to that in Fig. 2. The color bar shows the normalized quantity of OAM densities in units of *Ns*/*m* ^2^. The scale bar at the top in each figure is about 1 mm.

Figure 5 shows both the reconstructed and theoretical OAM density distributions. As we can see, the structures of the OAM density distributions resemble those of the intensity distribution in each case. An intuitive way to understand this is to recall that the OAM density originates from the cross product of the OFD with the radius vector with respect to the beam center, and this is given by

8 $${{\rm{j}}}_{{\rm{z}}}^{{\rm{orbital}}}=-\frac{{\varepsilon }_{{\rm{o}}}}{{\rm{2}}\omega }({|\alpha |}^{{\rm{2}}}\frac{\partial {\delta }_{{\rm{\alpha }}}}{\partial \varphi }+{|\beta |}^{{\rm{2}}}\frac{\partial {\delta }_{\beta }}{\partial \varphi })$$

where *ε*_*o*_ is the permittivity of free space, *ω* is the angular frequency of light, *k* is the wave number, *α* and *β* represent the horizontal and vertical components of light amplitudes, respectively, and *δ*_*α*_ and *δ*_*β*_ are the corresponding light phase of each component. Hence, referring back to Figs 2 and 4, there is OAM density provided that neither the intensity nor the local azimuthal phase gradient at the point in question are zero. Also, We can find that the structure of the OAM density and intensity is propagation-invariant in cases (a)–(d) and, in contrast, they are propagation-variant in cases (e) and (f). Supplementary Video 3 shows an animation of the OAM profiles evolving with propagation.

**Figure 6:**
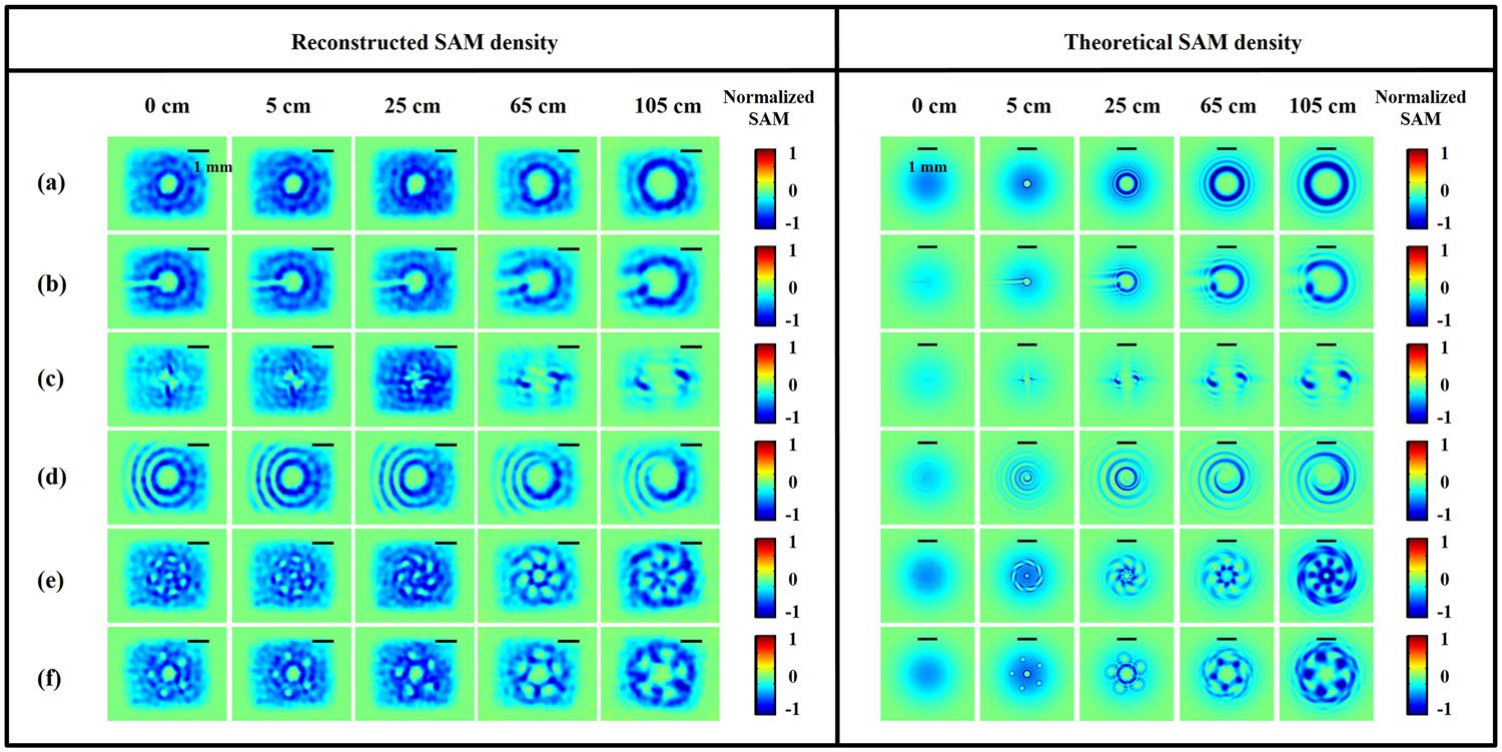
Spatial SAM density distributions of modulated beams on different cross sections. Left: DH reconstructed results; right: simulation results. Each case corresponds to that in Fig. 2. The color bar shows the normalized quantity of SAM densities in units of *Ns*/*m* ^2^. The scale bar at the top in each figure is about 1 mm.

Figure 6 shows both the reconstructed and theoretical SAM density distributions. As given in the Supplementary Equation (S33), the SAM density distribution mainly follows that of the Stokes parameters S_3_ of light. In addition, because the generated beam is LCP, it is expected that the SAM density structure is similar to that of the intensity distributions of each beam cross section, as we can see in the figure. Please take careful note that there is disagreement about how to define the handedness of a circularly polarized wave. Here, we adopt the convention used in the textbook^34^, in which the RCP (LCP) waves correspond to the positive (negative) sign of the SAM. Supplementary Video 4 shows an animation of the SAM profiles evolving with propagation.

**Figure 7:**
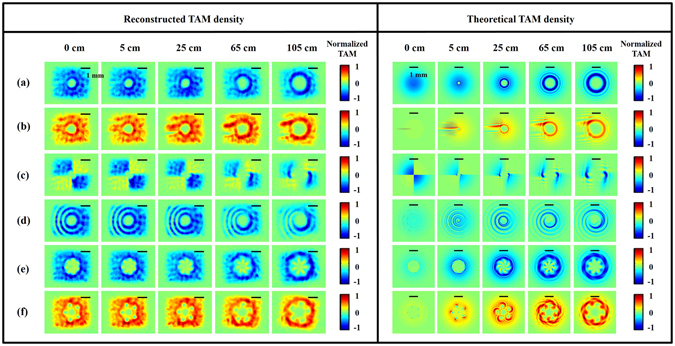
Spatial TAM density distributions of modulated beams on different cross sections. Left: DH reconstructed results; right: simulation results. Each case corresponds to that in Fig. 2. The color bar shows the normalized quantity of TAM densities in units of *Ns*/*m* ^2^. The scale bar at the top in each figure is about 1 mm.

Figure 7 shows both the reconstructed and theoretical TAM density distributions. Since the SAM charge of an LCP wave is always −1, the resulting local TAM density is wholly determined by the local TC on the beam. The actual local TC on the beam can be found from Fig. 4. For example, considering case (e) in Fig. 4, the actual TC in the interior region of light is +1 under the convention of ${e}^{j(\omega t-kz-\ell \varphi )}$. Therefore, we can clearly see from case (e) in Fig. 7 that the TAM density in the corresponding regions is close to zero. Similar arguments can be applied for the other cases.

## Discussion

In this study, we used a programmable imitated q-plate beam shaping system to experimentally demonstrate various beams carrying AM. When a circularly polarized wave is incident onto the system, the emerging beam reverses its polarization handedness and also obtains a phase factor. The phase factor can be engineered by displaying CGHs on the SLM. As such, it has more flexibility in controlling the OAM and TAM densities of light compared with the usual q-plate. In order to gain further insight on the generated fields, we combined DH with the beam modulation system to yield visualizations of the light properties, including the intensity, phase, SAM, and OAM along the beam propagation direction. We demonstrated that the local TAM density can be manipulated by changing the handedness of either the SAM or OAM, which results in their addition or subtraction from each other. Propagation-variant beams can be generated if CGHs used contain evenly distributed phase singularities around the holograms’ center; otherwise propagation-invariant beams can be generated. Moreover, we carried out a series of simulations and found that the smaller is the radial distance of the peripheral phase singularities, the faster is the profile evolution during light propagation without varying their steady state. It follows that we can obtain various propagation-variant beams by using other vortex structure holograms that are formed based on the extraction of phase profiles of the superposition between different LG modes^33^. Fields with changing OAM and intensity profiles during propagation promise to have interesting applications, such as the cross-linking of intensity-sensitive polymers for three-dimensional fabrications^35^ and OAM-driven micromachines^36^. To provide readers with the reliability of the proposed equivalent q-plate system, we also adopted a similar approach reported in work^37^ to measuring the helical modes of our modulated beams. Experimental results are given in the Supplementary Fig. S7.

Noise in the generated fields mainly originates from the fact of the pixelated structure of the SLM, which leads to the incomplete isolation of desired first-order from high-order spectrum contributions^24^. To remove the noise, we used a standard iris with a minimum aperture of about 0.8 mm placed at the focal plane in telescope 3. At the same time, it was also required that the spectrum of the encoded fields not be truncated by the iris. However, for situations in which the edge of the light pattern is unimportant, we can use an iris with a smaller aperture to further reduce the noise. The same situation also occurs in DH when generating a filtered hologram containing only the virtual image terms of the interference terms. To avoid truncating the high-frequency spectra of the interference terms, we made the transparent area of digital masks as large as possible. Here, we selected transparent mask radii of 50–60 pixels, whereas the total number of pixels in digital holograms is 3072, with zero-padding^19^. For detailed discussions, please refer to the supplementary information. Aberrations due to the misalignment of optical elements or SLM surface curvature may reduce the quality of modulated fields, and this can be compensated for by adding a compensated hologram to the SLM beforehand^38^.

## Electronic supplementary material

Supplementary information

Supplementary Video 1

Supplementary Video 2

Supplementary Video 3

Supplementary Video 4
