# Supplementary material for: Complex beam shaping based on an equivalent q-plate system and analysis of its properties using digital holography polarization imaging
Source: Sci Rep. 2017 Jun 5;7:2769. doi: 10.1038/s41598-017-02973-w (PMC5459860; doi:10.1038/s41598-017-02973-w)
Supplement: Supplementary file 1 — Supplementary information [file 41598_2017_2973_MOESM1_ESM.pdf]

Supplementary Information

Complex beam shaping based on an equivalent q-plate system and analysis of its properties using digital holography polarization imaging

Ching-Han Yang[1] and Andy Ying-Guey Fuh[1,2,3,*]

[1]*Department of Photonics, National Cheng Kung University, Tainan 701, Taiwan*

[2]*Department of Physics, National Cheng Kung University, Tainan 701, Taiwan*

[3]*Advanced Optoelectronic Technology Center, National Cheng Kung University, Tainan 701, Taiwan*

**Corresponding email: andyfuh@mail.ncku.edu.tw*

A. Theoretical description of the imitated q-plate beam shaping system

The imitated q-plate beam shaping system is illustrated in Fig. 1(b) of the main text. Here, optical elements P_2 and QWP_1 are excluded from our discussion, because they are simply used to adjust the object beam's polarization. The train of optical elements sandwiched between two crossed quarter-wave plates QWP_2 and QWP_4 constitute the imitated q-plate system, and therefore the Jones matrices of the imitated system can be obtained by matrix multiplication, as follows:

$$W_{imitated\ system} = W_{QWP4}(-45^{\circ}) \cdot W_{SLM,2} \cdot W_{4f} \cdot W_{SLM,1} \cdot W_{QWP2}(45^{\circ}) \qquad \text{(S1)}$$

where the subscript "SLM,1" and "SLM,2" indicate the first- and second-phase modulation, respectively, coming from different halves of the SLM. "QWP2" and "QWP4" are the two crossed quarter-wave plates, respectively, which we placed before and after the double phase modulation of light. The subscript "4f" indicates the reflective 4f imaging system in which we inserted QWP_3 to inverse two linear polarization components. Moreover, the Jones matrix of the 4f imaging system is given by:

$$W_{4f} = W_{QWP3}(-45^{\circ}) \cdot M_{M7} \cdot W_{QWP3}(45^{\circ}) = \begin{bmatrix} 0 & i \\ -i & 0 \end{bmatrix} \qquad \text{(S2)}$$

where the subscript "QWP3" indicates the quarter-wave plate QWP_3 and "M7" indicates the reflective mirror M_7. We note that careful consideration is necessary regarding the orientation of the slow (optical) axis of QWP_3. As shown in Fig. S1, due to mirror reflection, the incident beam passes QWP_3 with its slow axis oriented at 45° and the reflected beam oriented at −45° relative to the x-axis.

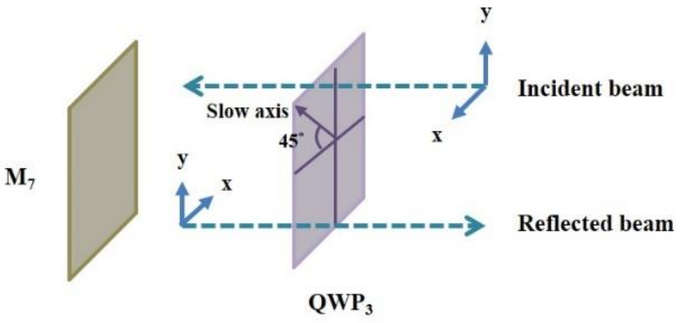

Figure S1. Illustration of the polarization rotation operation. M_7 (QWP_3) here is identical to M_7 (QWP_3) in Fig. 1(b) of the main text. Due to mirror reflection, the incident beam passes QWP_3 with its slow (optical) axis oriented at 45° and the reflected beam oriented at −45° relative to the x-axis.

We can show that $M_{4f} = \begin{bmatrix} 0 & i \\ -i & 0 \end{bmatrix}$ by substituting the following equations (S3)–(S5) into equation (S2).

$$M_{QWP3}(45°) = \frac{1}{\sqrt{2}}\begin{bmatrix} 1 & -i \\ -i & 1 \end{bmatrix} \tag{S3}$$

$$M_{QWP3}(-45°) = \frac{1}{\sqrt{2}}\begin{bmatrix} 1 & i \\ i & 1 \end{bmatrix} \tag{S4}$$

$$M_{M7} = \begin{bmatrix} -1 & 0 \\ 0 & 1 \end{bmatrix} \tag{S5}$$

The Jones matrix $M_{4f} = \begin{bmatrix} 0 & i \\ -i & 0 \end{bmatrix}$ is similar to a half-wave plate (HWP) with its slow axis oriented at 45° except that there is a minus sign in the second row and first column. This minus sign simply indicates that the reflected beam travels in the reverse direction, and therefore it is expected that the two orthogonal linear polarization components can be inversed when light passes through the system. For example, considering a beam with Jones vector $\begin{bmatrix} Ex \\ Ey \end{bmatrix}$ is incident onto the 4f imaging system, the Jones vector of the emerging beam from the 4f system becomes $i\begin{bmatrix} Ey \\ -Ex \end{bmatrix}$. To obtain the imitated system's Jones matrix, we can substitute equation (S2) and the following equations (S6)–(S9) into equation (S1).

$$M_{QWP2}(45°) = \frac{1}{\sqrt{2}}\begin{bmatrix} 1 & -i \\ -i & 1 \end{bmatrix} \tag{S6}$$

$$M_{QWP2}(\text{-}45°) = \frac{1}{\sqrt{2}}\begin{bmatrix} 1 & i \\ i & 1 \end{bmatrix} \tag{S7}$$

$$M_{SLM1,1} = \begin{bmatrix} e^{i \cdot p} & 0 \\ 0 & 1 \end{bmatrix} \tag{S8}$$

$$M_{SLM1,2} = \begin{bmatrix} e^{-i \cdot p} & 0 \\ 0 & 1 \end{bmatrix} \tag{S9}$$

where $M_{SLM,1}$ and $M_{SLM,2}$ have a similar form because the phase-only SLM is merely able to modulate the light whose polarization is parallel to the alignment of LC molecules, namely, in the x-direction. After performing some algebra, we can obtain the following:

$$M_{imitated\ system} = \begin{bmatrix} cos(p) & sin(p) \\ sin(p) & \text{-}cos(p) \end{bmatrix} \tag{S10}$$

As a result, the Jones matrix of the imitated system is similar to that of a typical q-plate [1]. It should be note that we have assumed an opposite sign between the first- and second-phase modulations in equations (S8) and (S9). Thus, the displayed CGH on area 2 of the SLM depends on that displayed on area 1.

Although the reflective matrix (S5) describes the situation where light is normally incident onto M7, it is also valid for the situation where light is obliquely incident onto the mirror at a small incident angle. For example, consider a beam obliquely incident onto a plane boundary from medium 1 into medium 2, then the reflection behavior of the beam can be described by the following equations:

$$\begin{aligned} r_s &= \frac{cos\theta - \sqrt{n^2 - sin^2\theta}}{cos\theta + \sqrt{n^2 - sin^2\theta}} \\ r_p &= \frac{-n^2 cos\theta + \sqrt{n^2 - sin^2\theta}}{n^2 cos\theta + \sqrt{n^2 - sin^2\theta}} \end{aligned} \tag{S11}$$

where r_p and r_s are respectively known as the reflection coefficients for the TM and TE modes, θ is the incident angle of light, n_1 and n_2 are the indices of refraction of the two media, and $n = \frac{n_2}{n_1}$ is the relative index of refraction. Here, we have assumed that both media are isotropic, linear, homogeneous, and lossless. As explained in the optics textbook [2], the TM mode can be considered as the horizontal component because its electric fields are parallel to the plane of incidence, while TE mode can be considered as the vertical component because its electric fields are orthogonal to the plane of incidence. Therefore, the Jones vector of the reflected beam can be obtained by

$$\begin{bmatrix} A' \\ B' \end{bmatrix} = \begin{bmatrix} -r_p & 0 \\ 0 & r_s \end{bmatrix} \begin{bmatrix} A \\ B \end{bmatrix}. \tag{S12}$$

where $\begin{bmatrix} A' \\ B' \end{bmatrix}$. and $\begin{bmatrix} A \\ B \end{bmatrix}$ are respectively the Jones vector for the reflected and incident beams. Because a typical reflective mirror is coated with a highly conductive metal film, thus it has very high value of permittivity in the visible light range, resulting in $n_2 >> n_1$, and therefore the relative index $n = \frac{n_2}{n_1}$ could be viewed as infinity. In this simple approximation, one can find the reflection matrix approximately becomes

$$\begin{bmatrix} -r_p & 0 \\ 0 & r_s \end{bmatrix} = -\begin{bmatrix} -1 & 0 \\ 0 & 1 \end{bmatrix} \tag{S13}$$

Notably, above equation is identical to equation (S5) except for a minus sign in front of the matrix, which is unimportant in the Jones formulism calculation. As for wave plate QWP3, some phase retardation error may result from the light obliquely incident on it, and this effect could be further reduced by using a high quality wave plate in which the phase retardation is less dependent on the incident angle of light.

B. Theoretical description of the reconstruction of modulated beams

In the experiment, we compute the vertical and horizontal components of the modulated beam on different propagation planes based on the angular spectrum method [3]. As an example, we assume that the corrected holograms at the hologram (CCD) plane is given by $O_i(x,y,z=0)$, so its corresponding angular spectrum can be obtained by taking its Fourier transform, as follows:

$$A_{o,i}(k_x,k_y;z=0) = \iint O_i(x,y,z=0) exp\,[-j \cdot (k_x x + k_y y)]\; dxdy \tag{S14}$$

where the subscript "i" represents the x or y component of light, k_x and k_y represent the spatial frequencies in the x and y directions, respectively, and the hologram plane is labeled with z = 0. Please take careful note that the plane z = 0 is referred as the modulated beam source plane 2* in the main text. Equation (S14) is therefore a decomposition of the field $O_i(x,y,z=0)$ into a collection of plane waves having different wave vectors (k_x,k_y,k_z), where $k_z = \sqrt{k^2 - k_x^2 - k_y^2}$, and $k = 2\pi/\lambda$. After propagating over a distance d, each plane wave will acquire an additional phase factor $exp(jk_z d)$, and thus the new angular spectrum $A_{o,i}(k_x,k_y;z=d)$ at the plane z = d can be written as follows:

$$A_{o,i}(k_x,k_y;z=d) = A_{o,i}(k_x,k_y;z=0) \cdot exp(jk_z d) \tag{S15}$$

The complex amplitude of the propagating field $O_i(x, y, z = d)$ can be obtained by taking the inverse Fourier transform of equation (S15); that is

$$O_i(x,y;z=d) = \iint A_{o,i}(k_x,k_y;z=d) exp\,[j \cdot (k_x x + k_y y)]\, dxdy \tag{S16}$$

We note that the sign of d can be negative or positive depending on whether the reconstruction plane is placed behind or in front of the hologram plane. We can calculate the field's intensity and phase at plane z = d by the following equations, respectively:

$$I_i(x,y;z=d) = \left|O_i(x,y;z=d)\right|^2 \tag{S17}$$

$$\phi_i(x, y; z=d) = \arg\{\mathrm{O}_i(x, y; z=d)\} \tag{S18}$$

where operator "arg" denotes the argument of equation (S16). In addition, we can find the Stokes parameters by the following definitions:

$$S_0 \equiv O_x^2 + O_y^2 \tag{S19}$$

$$S_1 \equiv O_x^2 - O_y^2 \tag{S20}$$

$$S_2 \equiv O_x O_y^* + O_y O_x^* \tag{S21}$$

$$S_3 \equiv i \cdot (O_x O_y^* - O_y O_x^*) \tag{S22}$$

To further compute the orbital angular momentum (OAM) and spin angular momentum (SAM) densities of light, here we briefly outline the approach that we used. For simplicity, we drop the notation z = d in the following discussion. As explained in [4], a convenient representation of vectorial fields can be achieved by the Lorenz gauge using the complex vector potential, as follows:

$$\vec{A}_c(x,y,t) = \frac{i}{\omega}\left(\alpha(x,y)\,\hat{e}_x + \beta(x,y)\,\hat{e}_y\right) e^{-ikz} \tag{S23}$$

where the subscript “c” indicates complex fields, α and β are identical to O_x and O_y, respectively, which we have addressed in previous discussions, ω is the angular frequency of light, and k is its wave number. We can show that the complex amplitudes of the electric and magnetic field components of light under paraxial approximation are as follows:

$$\begin{aligned}\vec{B}_c(x,y) &= \nabla\times\vec{A}(x,y)\\ &= -\frac{k}{\omega}\left(\beta(x,y)\hat{e}_x-\alpha(x,y)\hat{e}_y+\frac{i}{k}\left(\frac{\partial}{\partial y}\alpha(x,y)-\frac{\partial}{\partial x}\beta(x,y)\right)\hat{e}_z\right)\end{aligned}\tag{S24}$$

$$\begin{aligned}\vec{E}_c(x,y) &= -\frac{ic^2}{\omega}\nabla\times\vec{B}_c(x,y)\\ &= \alpha(x,y)\hat{e}_x+\beta(x,y)\hat{e}_y-\frac{i}{k}\left(\frac{\partial}{\partial x}\alpha(x,y)+\frac{\partial}{\partial y}\beta(x,y)\right)\hat{e}_z\end{aligned}\tag{S25}$$

where c in the equations is the light speed. As it is well known, the time-averaged linear momentum density, denoted as $\vec{p}_{av}$, is as follows:

$$\vec{p}_{av}=\varepsilon_o\left\langle\vec{E}_{real}\times\vec{B}_{real}\right\rangle\tag{S26}$$

where $\vec{E}_{real}=\frac{1}{2}\left(\vec{E}_c+\vec{E}_c^*\right)$ and $\vec{\mathrm{B}}_{real}=\frac{1}{2}\left(\vec{\mathrm{B}}_c+\vec{\mathrm{B}}_c^*\right)$ are real physical fields, and the subscript “av” represents the time-averaged. By substituting equations (S24) and (S25) into equation (S26), we can find the time-averaged transverse and longitudinal components of $\vec{p}_{av}$, respectively, as follows:

$$\vec{p}_\perp(x,y)=\frac{i\varepsilon_o}{4\omega}\left(\alpha^*\nabla\alpha+\beta^*\nabla\beta-\alpha\nabla\alpha^*-\beta\nabla\beta^*+\nabla\times[(\alpha^*\beta-\alpha\beta^*)\hat{z}]\right)\tag{S27}$$

$$p_z(x,y)=\frac{k\varepsilon_o}{2\omega}\left(|\alpha|^2+|\beta|^2\right)\tag{S28}$$

where ε_o is the permittivity of free space and $\nabla\equiv\begin{pmatrix}\partial/\partial x\\ \partial/\partial y\end{pmatrix}$ is the transverse gradient operator. For simplicity, we have dropped the subscript “av” in the discussions. We can find the φ-component of the transverse component $\vec{p}_\perp$ as follows:

$$p_\varphi(x,y)=\frac{i\varepsilon_o}{4\omega}\frac{1}{r}\left(\alpha^*\frac{\partial}{\partial\varphi}\alpha+\beta^*\frac{\partial}{\partial\varphi}\beta-\alpha\frac{\partial}{\partial\varphi}\alpha^*-\beta\frac{\partial}{\partial\varphi}\beta^*-r\left(\frac{\partial}{\partial r}(\alpha^*\beta-\alpha\beta^*)\right)\right)\tag{S29}$$

The product of p_φ with r (radial distance with respect to the beam center) gives the z component of the time-averaged total angular momentum (TAM) density, denoted as $j_z^{total} = \langle \vec{r} \times \vec{p} \rangle_z = rp_\phi$. Specifically, it follows that TAM can be divided into SAM and OAM densities, as given in equations (S31) and (S32), respectively:

$$j_z^{total}(x,y) = j_z^{spin} + j_z^{orbital} \tag{S30}$$

$$j_z^{spin} = \frac{-i\varepsilon_o}{4\omega} r \frac{\partial}{\partial r}\left(\alpha^* \beta - \alpha \beta^*\right) \tag{S31}$$

$$\begin{aligned} j_z^{orbital} &= \frac{i\varepsilon_o}{4\omega}\left(\alpha^* \frac{\partial}{\partial \varphi}\alpha + \beta^* \frac{\partial}{\partial \varphi}\beta - \alpha \frac{\partial}{\partial \varphi}\alpha^* - \beta \frac{\partial}{\partial \varphi}\beta^*\right) \\ &= \frac{-\varepsilon_o}{2\omega}\left(|\alpha|^2 \frac{\partial \delta_\alpha}{\partial \varphi} + |\beta|^2 \frac{\partial \delta_\beta}{\partial \varphi}\right) \end{aligned} \tag{S32}$$

where we have used equations $\alpha = |\alpha| \cdot e^{j\delta_\alpha}$ and $\beta = |\beta| \cdot e^{j\delta_\beta}$ in the last expression. As we can find, equation (S31) relates to the mixtures of light's vertical and horizontal components and thus is associated with the polarization, which yields the SAM density. Equation (S32) relates to the azimuthal phase gradient of each vertical and horizontal component and thus is associated with the spatial phase distribution, which yields the OAM density. However, we note that due to a peculiar property of SAM, the actual SAM density should also include the contribution of energy circulation along the boundary within every microscopic cell on the beam cross section [5]. After taking into account the boundary contribution, the correct formula for SAM density is given by the following:

$$j_z^{spin} = \frac{i\varepsilon_o}{2\omega}\left(\alpha \beta^* - \alpha^* \beta\right) = \frac{\varepsilon_o}{2\omega} S_3 \tag{S33}$$

With reference to $S_3 \equiv i\left(\alpha\beta^* - \alpha^*\beta\right)$, we find that the SAM density distribution resembles that of Stokes parameters S_3, which is also mentioned in earlier work [6].

Of course, we could have a similar discussion from the perspective of the Poynting vector (energy flow density), since the Poynting vector and linear momentum density differ only by a constant factor c^2 in free space. Following equations (S27) and (S28), the time-averaged transverse and longitudinal energy flow densities are given by the following:

$$\vec{S}_\perp(x,y) = c^2 \cdot \vec{P}_\perp = \vec{S}_\perp^{spin} + \vec{S}_\perp^{orbital} \tag{S34}$$

$$S_z(x,y) = c^2 \cdot P_z \tag{S35}$$

$$\vec{S}_{\perp}^{orbital}(x,y) = \frac{i\varepsilon_o c^2}{4\omega}\left(\alpha^*\nabla\alpha + \beta^*\nabla\beta - \alpha\nabla\alpha^* - \beta\nabla\beta^*\right) \tag{S36}$$

$$\vec{S}_{\perp}^{spin}(x,y) = \frac{i\varepsilon_o c^2}{4\omega}\left(\nabla \times [(\alpha^*\beta - \alpha\beta^*)\hat{z}]\right) \tag{S37}$$

where, the total transverse energy flow density (TFD) $\vec{S}_{\perp}$ is decomposed into its spin $\vec{S}_{\perp}^{spin}$ and orbital $\vec{S}_{\perp}^{orbital}$ aspects, respectively. Specifically, the former is called the "spin flow density (SFD)," while the latter is the "orbital flow density (OFD)" [4]. As expected, the cross product of SFD (OFD) with the radius vector contributes to the SAM (OAM) density. In our experiment, we reconstructed the OFD, SAM, and OAM density distributions of a propagating field at each plane parallel to the hologram plane by DH using equations (S36), (S31), and (S32), where α and β are replaced, respectively, by $\mathrm{O_x}$ and $\mathrm{O_y}$, which are given in equation (S16). Supplementary Videos 1, 2, 3, and 4 online respectively shows an animation of the intensity, phase, OAM, and SAM profiles evolving with propagation.

C. Detailed information on experimentally recorded holograms and the filtering process

Figures S2(a)–S2(f) give the digital holograms recorded by a CCD camera, where each case corresponds to that in the Fig. 2 captions. Figures S2(g) and S2(h) respectively give the intensity distributions of two reference beams that are vertically and horizontally polarized, respectively. Figure S2(i) gives the reference hologram, which was used to compensate the tilted phase factor introduced by the off-axis holography. Figure S2(j) gives a larger view of the central portion of Fig. S2(e), which permits us to see two orthogonally distributed gratings corresponding to the interference of the modulated beam with two orthogonally polarized reference beams. In order to avoid fringing in the reconstruction plane, we apodized these holograms by a smooth window function characterized by a transmission that does not change abruptly from zero to unity at the edge of the aperture. Otherwise, there are spatial fluctuations in the vicinity of the marginal areas of fields, as shown in Figure S3.

Figures S4(a) and S4(b), respectively, correspond to the signal (e) and reference (i) hologram spectra in Fig. S2, with 3072 × 3072 pixels after zero-padding. Figures S4(c) and S4(d) show the filtering process performed on Fig. S4(a) in order to simultaneously extract the virtual image terms $O_{x,s}R_x^*$ and $O_{y,s}R_x^*$ from the spectrum by multiplying a transparent digital mask with a radius of 50 pixels on Fig. S4(a). In this figure, $O_{i,s}$ ($O_{i,b}$) represents the x or y component of modulated (blank) object beams, and R_i denotes the x- or y-polarized reference beam. The same filtering procedure is also performed on the reference hologram spectrum of Fig. S4(b). The inverse Fourier transform of the filtered spectra results in two filtered holograms containing only the virtual

images of interference terms, as given in equation (4) in the main text. Subsequently, the tilted phase factor due to off-axis holography is compensated by using the reference conjugated hologram method. Then, corrected holograms containing only the information of the x or y component of the modulated beam can be obtained from equation (7) in the main text.

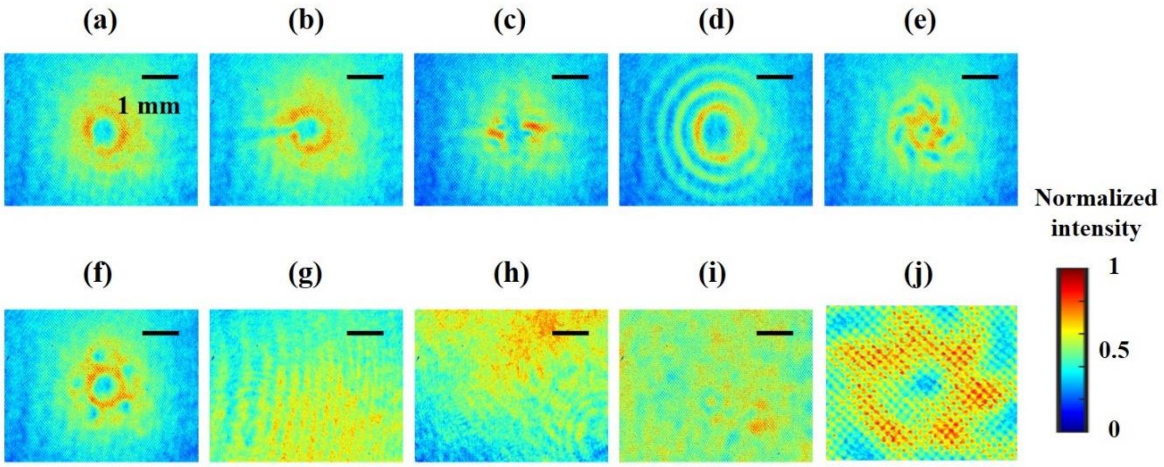

Figure S2. Digital holograms recorded by a CCD camera. Cases (a)–(f) correspond to those in Fig. 2 in the main text, (g) and (h) show the intensity distributions of vertically and horizontally polarized reference beams, respectively, (i) shows the reference hologram, (j) shows the larger view of the central portion of (e). The scale bar on the right-top corner in each figure is about 1 mm.

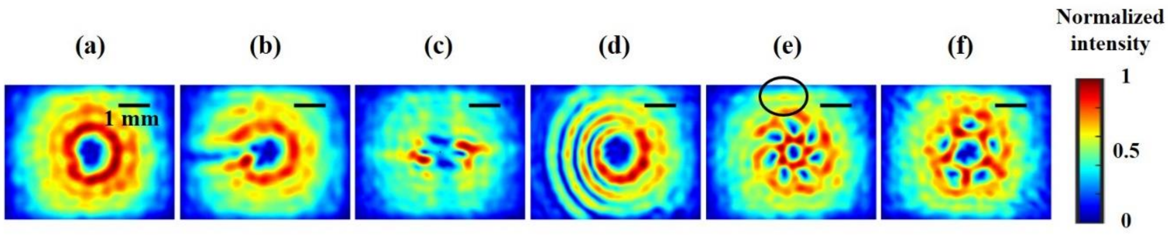

Figure S3. DH reconstructed intensities over the propagation distance 65 cm from the modulated beam source plane 2*. Cases (a)–(f) correspond to those in Fig. 2 in the main text. The intensity fluctuation induced by the edge of the hologram aperture can be clearly seen in the margin areas of the figures, for example, in the black circle marked at the top of (e). The scale bar on the right-top corner in each figure is about 1 mm.

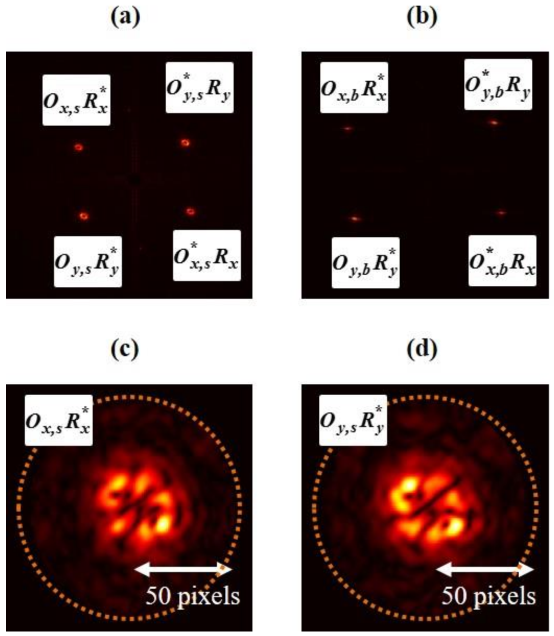

Figure S4. Hologram spectra and the associated filtering process. (a) Spectrum of Fig. S2(e), (b) Spectrum of Fig. S2(i), (c) and (d) correspond to the filtering process performed on (a) in order to extract the x and y components of the interference terms, respectively, where the radii of two transparent masks (dashed lines) which are multiplied on (a) range from 50 to 60 pixels, depending on the maximum band width of the interference terms.

D. Detailed information on how to accurately align the beam shaping system

The simplest way of accurately aligning the experimental setup is to respectively encode two helical phase modes onto the x and y components of a linearly polarized incident beam. Then, the modulated beam is sent to pass through a polarizer with its transmission axis oriented at x- or y-axis. In addition, we use a CCD camera to record the two donut-shape intensities of x- and y-polarization components of the modulated beam. Next, we arrange the optical elements of L5, and M7, such that they roughly constitute a reflective 4f imaging system. Finally, we carefully adjust the elements of L5 and M7 so that both the diameters and the central dark points of the two donut-shape intensities can be completely overlapped with each other. A more detailed description of the calibration process is given as follows.

As shown in Fig. S5, we replaced QWP2 used in Fig. 1(b) in the manuscript by a polarizer, denoted as P3, with its transmission axis oriented at 45° with respect to the x-axis to produce a linearly polarized beam, and we replaced QWP4 used in Fig. 1(b) in the manuscript by an analyzer, denoted as P4. The CCD camera was placed just behind the analyzer P4. At the same time, two blazed gratings as mentioned in the manuscript were respectively added to two designed holograms with $e^{i\phi}$ phase distributions, and then they were displayed on each half of the SLM. In this way, the Jones vector of the incident beam after passing through polarizer P3 can be given by the following:

$$\vec{E}_{in} = \begin{bmatrix} E_x \\ E_y \end{bmatrix} = \begin{bmatrix} cos45^\circ \\ sin45^\circ \end{bmatrix} = \frac{1}{\sqrt{2}} \begin{bmatrix} 1 \\ 1 \end{bmatrix} \tag{S38}$$

During the first modulation, the x component of the electric field is encoded by a phase factor of $e^{i\phi}$ while the y component is not. Therefore, when the beam is reflected off the area 1 of the SLM surface, the Jones vector is given by:

$$\vec{E}1 = \frac{1}{\sqrt{2}} \begin{bmatrix} e^{i\phi} \\ 1 \end{bmatrix} \tag{S39}$$

where the subscript "1" means the first modulation. As mentioned before, the reflective 4f system was used to inverse the x and y components of light, and the Jones matrix of the 4f system is given in equation (S2). Therefore, after the beam passing through the 4f imaging system, the Jones vector becomes

$$\vec{E}1' = \frac{1}{\sqrt{2}} \begin{bmatrix} 0 & i \\ -i & 0 \end{bmatrix} \begin{bmatrix} e^{i\phi} \\ 1 \end{bmatrix} = \frac{i}{\sqrt{2}} \begin{bmatrix} 1 \\ -e^{i\phi} \end{bmatrix} \tag{S40}$$

During the second modulation, the x component is again encoded, leaving the y component unaffected. Thus, the Jones vector of the reflected beam is given as follows

$$\vec{E}2 = \frac{i}{\sqrt{2}} \begin{bmatrix} e^{i\phi} \\ -e^{i\phi} \end{bmatrix} \tag{S41}$$

where the subscript "2" means the second modulation. Next, an analyzer P4 is used to select the x- or y- component of the modulated beam. Finally, we carefully aligned the spatial positions of M7 and L5 and adjusted the tilted angle of M7 so that both the donut-shape intensities of the x- and y-components could be completely overlapped as precise as possible.

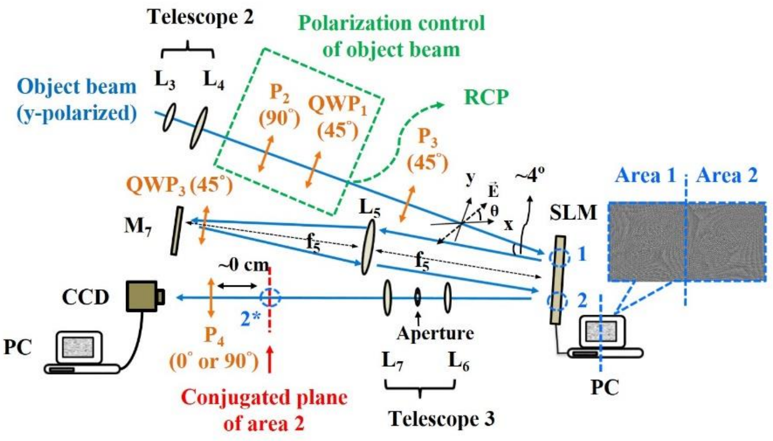

Figure S5. Detailed calibration process of the beam shaping system. The setup is similar to Fig. 1(b) shown in the manuscript except that both the QWP_2 and QWP_4 wave plates are respectively replaced by a polarizer P_3 and an analyzer P_4. The transmission axis of the P_3 is fixed at 45° with respective to the x-axis, while that of P_4 is chosen to be oriented at either the x-axis or y-axis. Two designed blazed helical phase holograms are respectively displayed on each half of the SLM.

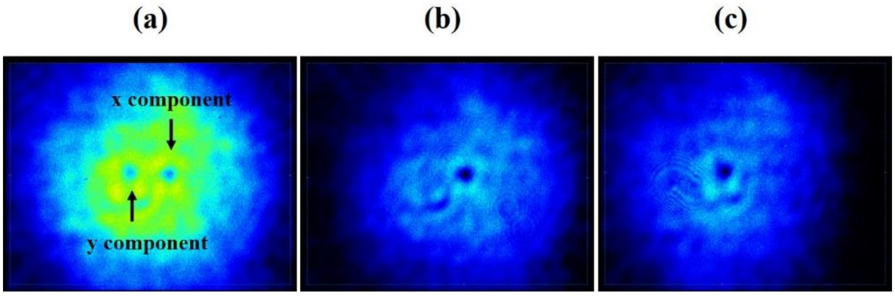

Figure S6. Calibration process. (a) Two orthogonally polarized donut-shape intensities are separately distributed from each other without the modulated beam passes through P_4, (b) image of the x polarization component when the modulated beam passes through P_4 with its transmission axis aligned in the x-axis, (c) image of the y polarization component when light passes through P_4 with its transmission axis aligned in the y-axis.

E. Demonstration of the helical phase front using a typical interferometer

To provide readers with the reliability of the proposed equivalent q-plate system, we also adopted a similar approach reported in work [7] to measuring the helical modes of our modulated beams. The experimental setup is similar to that of Fig. 1 in our manuscript except that we placed another polarizer with its transmission axis oriented at the x-axis behind the QWP4 wave plate (Fig. 1(b)), and the y-polarized reference beam was blocked while the x-polarized reference beam was not (Fig. 1(a)). Then, we used a CCD camera to record the interference patterns made by both the x-polarized modulated beam and the x-polarized reference beam. Figure S7 displays the corresponding experimental results, where the pairs of (a) and (e), (b) and (f), and (c) and (g) respectively correspond to the results obtained by displaying holograms with TC equal to 1, 2, and 3 on the SLM, and the pair

of (d) and (h) corresponds to the result obtained by displaying the same holograms as that used in Fig. 2(d) in our manuscript on the SLM. The first row of Fig. S7 are the interference patterns in which the x-polarized reference beam is approximately planar; while the second row of the figure are the interference patterns in which the x-polarized reference beam is approximately spherical, as obtained by inserting a lens in its path. As it can be seen, for the case of planar-wave reference beam, these interference patterns feature the fork-like patterns; for the case of spherical-wave reference beam, these interference patterns feature the spiral-like patterns. These measured results agree with those obtained by typical q-plates [7] and meta-q-plates [8].

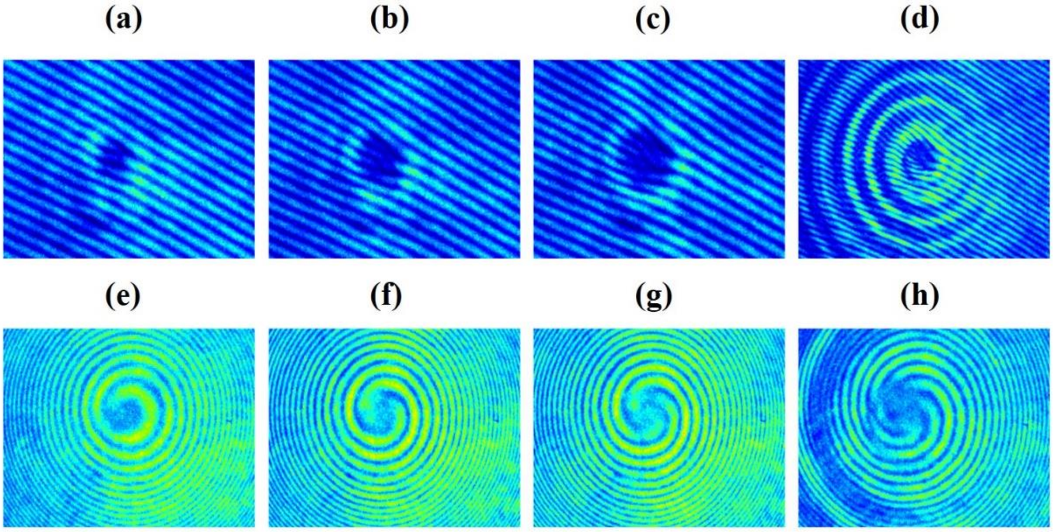

Figure S7. Measured interference patterns. The first row corresponds to the planar-wave reference beam, while the second row corresponds to the spherical-wave reference beam. The pairs of (a) and (e), (b) and (f), and (c) and (g) respectively correspond to the results obtained by displaying holograms with TC equal to 1, 2, and 3 on the SLM, and the pair of (d) and (h) corresponds to the result obtained by displayed the same holograms as that used in Fig. 2(d) in our manuscript on the SLM.

F. Supplementary Videos 1, 2, 3, and 4 online respectively shows an animation of the intensity, phase, OAM, and SAM profiles evolving with propagation.

References

1. L. Marrucci, C. Manzo, and D. Paparo, "Optical Spin-to-Orbital Angular Momentum Conversion in Inhomogeneous Anisotropic Media," Physical Review Letters **96**, 163905 (2006).
2. G. R. Fowles, "Introduction to Modern Optics (2nd Edition)," (Dover Publications), p. 52.
3. M. K. Kim, "Principles and techniques of digital holographic microscopy," PHOTOE, 018005-018005-018050 (2010).
4. A. Y. Bekshaev and M. S. Soskin, "Transverse energy flows in vectorial fields of paraxial beams with singularities," Optics Communications **271**, 332-348 (2007).
5. A. Bekshaev, "Spin angular momentum of inhomogeneous and transversely limited light beams," in 2006), 625407-625407-625408.
6. C. Rui-Pin, Z. Li-Xin, C. Khian-Hooi, G. Bing, Z. Guoquan, and Z. Tingyu, "Effect of a spiral phase on a vector optical field with hybrid polarization states," Journal of Optics **17**, 065605 (2015).
7. L. Marrucci, "Generation of Helical Modes of Light by Spin-to-Orbital Angular Momentum Conversion in Inhomogeneous Liquid Crystals," Molecular Crystals and Liquid Crystals **488**, 148-162 (2008).
8. W. Ji, C.-H. Lee, P. Chen, W. Hu, Y. Ming, L. Zhang, T.-H. Lin, V. Chigrinov, and Y.-Q. Lu, "Meta-q-plate for complex beam shaping," Scientific Reports **6**, 25528 (2016).
